# Caffeine Biotransformation in Zebrafish Larvae: Integrated LC-MS/MS Quantification and FTIR Spatial Mapping

**DOI:** 10.3390/molecules31071095

**Published:** 2026-03-26

**Authors:** Adrian Bartoszek, Anna Kozub-Pędrak, Alicja Wielgosz, Agata Sumara, Kacper Przykaza, Grzegorz Kalisz, Anna Stachniuk

**Affiliations:** Department of Bioanalytics, Medical University of Lublin, Kazimierza Jaczewskiego St. 8b, 20090 Lublin, Poland; 51112@umlub.edu.pl (A.K.-P.); alicja.wielgosz@umlub.edu.pl (A.W.); agata.sumara@umlub.edu.pl (A.S.); kacper.przykaza@umlub.edu.pl (K.P.); anna.stachniuk@umlub.edu.pl (A.S.)

**Keywords:** caffeine, caffeine metabolism, zebrafish larvae, mass spectrometry, FTIR

## Abstract

Caffeine (CAF) is one of the most widely consumed psychoactive substances worldwide. It is primarily metabolized to paraxanthine (PAR), theobromine (THR), and theophylline (THY). While CAF metabolism has been extensively characterized in humans and rodent models, corresponding data in zebrafish (*Danio rerio*) larvae remain limited. This study provides a comprehensive characterization of CAF biotransformation in zebrafish larvae using validated LC-QQQ-MS for quantitative metabolite profiling and FTIR imaging for spatially resolved tissue distribution mapping. Zebrafish larvae at 4 days post fertilization (dpf) were exposed to CAF at concentrations of 15, 25, and 50 mg/L for 18 h. The LC–MS/MS analysis demonstrated dose-dependent conversion of CAF to PAR (up to 4.54%), THR (up to 1.32%), and THY (up to 0.27%). The PAR/CAF and THR/CAF ratios increased as CAF concentration rose, while the THY/CAF ratio declined. In parallel, FTIR imaging was applied to visualize the spatial distribution of CAF and selected metabolites in larval tissue sections, confirming the presence of CAF, PAR, and THR and revealing their localization within the tissue. However, THY was not detected by this method. Metabolite localization differed across larval tissues, indicating compartmentalized metabolic processes. This study provides the first comprehensive characterization of CAF metabolism in zebrafish larvae, revealing low but detectable CYP450-mediated metabolic activity prior to full hepatic maturation. The findings support the utility of zebrafish larvae as a model for developmental pharmacokinetic studies.

## 1. Introduction

Caffeine (CAF) remains one of the most extensively ingested stimulants worldwide. This compound falls within the category of compounds known as purine alkaloids [1]. CAF is present in beverages commonly consumed daily, such as coffee and tea [2]. Various botanical sources contain CAF within their seeds, fruits, and foliage. Notable examples include cacao beans, found in chocolate; yerba mate leaves, used for herbal tea; and guarana berries incorporated into various beverages and supplements [3]. For centuries, coffee and tea have been integral to cultural customs and social interactions. Moreover, individuals utilize coffee to enhance alertness and productivity in their daily activities [3]. In North America, approximately 90% of the population consumes beverages containing CAF [4]. Furthermore, van Dam et al. (2020) highlight that although it exhibits numerous health benefits, current evidence does not justify recommending CAF or coffee consumption for disease prevention [3]. However, they suggest that moderate intake of coffee or tea can be incorporated into the lifestyle of adults without specific health conditions, excluding pregnant or lactating individuals [3]. On the contrary, CAF is associated with adverse effects related to excessive CAF consumption. These effects include anxiety, restlessness, nervousness, dysphoria, insomnia, excitement, psychomotor agitation, and rambling speech. It is important to note that these symptoms are not limited to these and can vary from person to person [3,5]. Moreover, it is noteworthy that CAF undergoes hepatic metabolism primarily via demethylation, a process facilitated predominantly by the cytochrome P450 enzyme family, notably CYP1A2, accounting for approximately 95% of the conversion. This enzymatic process leads to the production of CAF’s primary metabolites, including PAR, THR, and THY, which undergo subsequent metabolism to uric acid and are ultimately excreted via urine [6]. Caffeine and its principal derivatives exert various effects on physiological functions. CAF itself enhances the basal metabolic rate and acts as a mild stimulant for the central nervous system, heart, and smooth muscles. Moreover, PAR, the most abundant and biologically active metabolite, acts as a central nervous system stimulant with comparable activity to CAF but tends to have lower toxicity and fewer anxiety-inducing effects. THR also exerts stimulating effects, although they are generally milder than CAF. Lastly, THY is a widely used bronchodilator with a limited therapeutic range in the bloodstream [7].

Zebrafish (*Danio rerio*) has become an established in vivo model in biomedical research, largely due to its genetic similarity to humans (~70%) and broad applicability across different experimental settings [8]. This model offers several advantages, including low maintenance requirements, rapid development, and fewer ethical constraints compared to mammalian systems. In accordance with the principles of the 3Rs (Replacement, Reduction, and Refinement), the use of zebrafish larvae allows for a reduction in animal usage while maintaining the relevance of biological observations [9]. At early developmental stages (4–5 days post fertilization (dpf)), zebrafish larvae already exhibit functional physiological processes, including absorption and metabolic activity, despite incomplete hepatic maturation. In addition, their small size and optical transparency, combined with the possibility of high-throughput experimental design, support their application in pharmacokinetic and toxicological studies [10].

As of now, no investigations have been conducted on the primary three CAF metabolites in zebrafish embryos, larvae, or adults. In fish studies, tested compounds are usually dissolved in the medium. The investigated dose is often described as the concentration in the medium rather than in larvae. The relationship between the external concentration of the exogenous compound in the medium and its internal exposure is crucial for accurately interpreting the observed results, as it is the internal concentration that drives pharmacological and toxicological effects. Ignoring this critical issue has been reported to result in unfavorable outcomes in drug research [11]. Therefore, research on CAF should involve quantifying drugs and their metabolites to interpret the data obtained accurately. Furthermore, assessing the internal concentration based on the compound’s physicochemical properties has proven to be quite challenging [12]. Another significant challenge is the small size of the larvae, necessitating the use of highly sensitive methods. It should be emphasized that compounds’ absorption and elimination depend on the larvae’s age [13]. In zebrafish larvae, xenobiotics present in the exposure medium are primarily absorbed via passive diffusion through the skin and gills. Due to their small size and high surface-to-volume ratio, uptake of small molecules is generally efficient, although internal concentrations may differ from nominal exposure levels [13].

In recent years, the integration of multiple analytical techniques has become increasingly common in biochemical and metabolic research, providing a more comprehensive understanding of complex biological systems. Liquid chromatography–mass spectrometry (LC-MS) and Fourier-transform infrared spectroscopy (FTIR) are two complementary methods that have proven invaluable in such studies. LC-MS combines the separation power of LC with the molecular specificity of MS, enabling the simultaneous detection and quantification of multiple compounds with high sensitivity. This makes it a gold-standard method for metabolite profiling and pharmacokinetic studies in complex biological matrices [14]. FTIR spectroscopy, on the other hand, offers a rapid, non-invasive approach to identifying and analyzing functional groups within molecules based on their vibrational properties. When combined with imaging, FTIR allows for the spatial mapping of molecular distributions in tissues, offering valuable insights into structural and functional dynamics [15].

**Figure 1:**
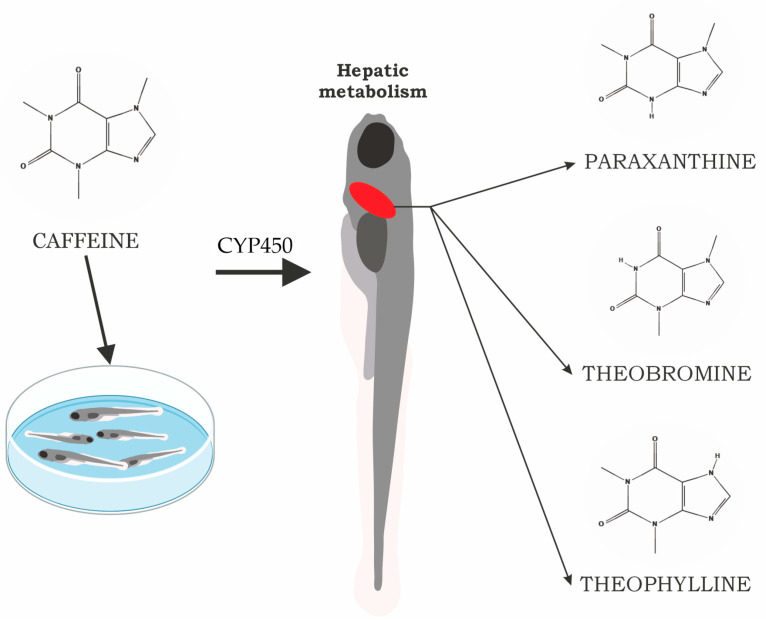
Schematic representation of the in vivo pathways of CAF metabolism in zebrafish larvae leading to the formation of the major metabolites PAR, THR and THY. Schematic drawings were obtained from Scidraw.io.

The aim of this study was to investigate CAF biotransformation in zebrafish larvae, with particular focus on its primary metabolites PAR, THR, and THY (Figure 1). Quantitative analysis was performed using LC-MS/MS, while FTIR spectroscopy was applied to confirm the presence of the compounds and provide spatial information on their distribution within larval tissues.

## 2. Results

### 2.1. Quantification of CAF, PAR, THR and THY Levels in the Zebrafish Larvae

**Figure 2:**
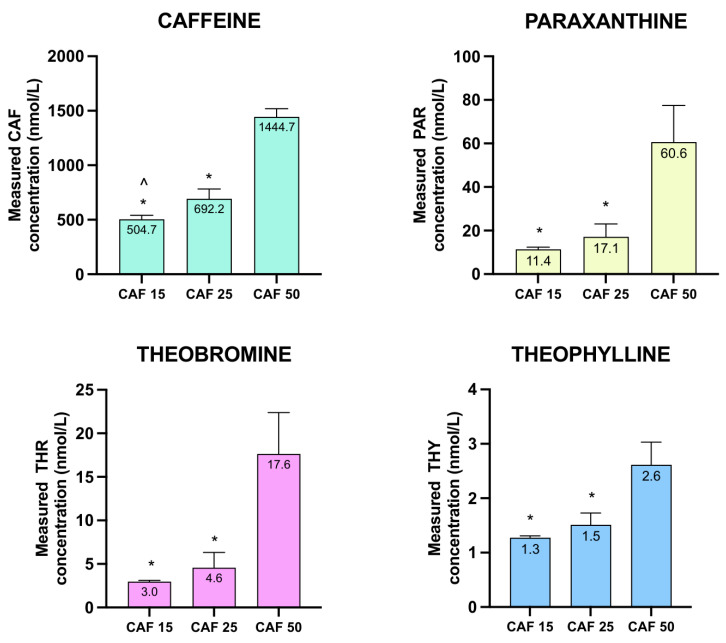
Contents of CAF, PAR, THR, and THY for larvae treated with CAF. CAF concentration applied at 15, 25, and 50 mg/L. * *p* < 0.05, as compared to CAF 50 mg/L group, ^ *p* < 0.05, as compared to CAF 25 mg/L group.

The zebrafish larvae were treated with three different concentrations of CAF: 15 mg/L, 25 mg/L, and 50 mg/L. Firstly, the contents of CAF and its three metabolites were measured (Figure 2). CAF concentrations differed among all investigated solutions, measuring 504.7, 692.2 and 1444.7 nmol/L, respectively. The contents of each metabolite, namely PAR, THR, and THY, were significantly higher in the group administered 50 mg/L CAF compared to the groups receiving lower concentrations of 25 and 15 mg/L (65.3 vs. 18.4 and 12.3 nmol/L; 19 vs. 4.9 and 3.2 nmol/L; 2.8 vs. 1.6 and 1.4 nmol/L, respectively). However, there was no significant difference in metabolite concentration between the group administered 15 and 25 mg/L CAF (0.692 vs. 0.505 nmol/L; 0.018 vs. 0.012 nmol/L; 0.005 vs. 0.003 nmol/L, respectively) (Figure 2). Importantly, the absence of significant differences in metabolite concentrations between 15 and 25 mg/L groups, when contrasted with the marked increase at 50 mg/L, suggests a non-linear activation of CAF biotransformation pathways in zebrafish larvae.

The LC-QQQ-MS method demonstrated high sensitivity and satisfactory precision for all four analytes. The limits of detection (LOD) and quantification (LOQ) were: CAF 5.358 and 17.859 nmol/L; PAR 0.430 and 1.434 nmol/L; THR 1.118 and 3.725 nmol/L; THY 0.389 and 1.298 nmol/L, respectively (Table 1). Precision, expressed as relative standard deviation (RSD, %), did not exceed 9.01% at the lowest and 4.26% at the highest calibration concentration, confirming the method’s reproducibility across the working range. The low LOQ values for PAR, THR, and THY were critical for the reliable quantification of metabolites present at trace levels, especially THY, whose concentrations (1.4–2.8 nmol/L) were close to but consistently above the LOQ threshold.

### 2.2. Metabolites-to-CAF Ratio

**Figure 3:**
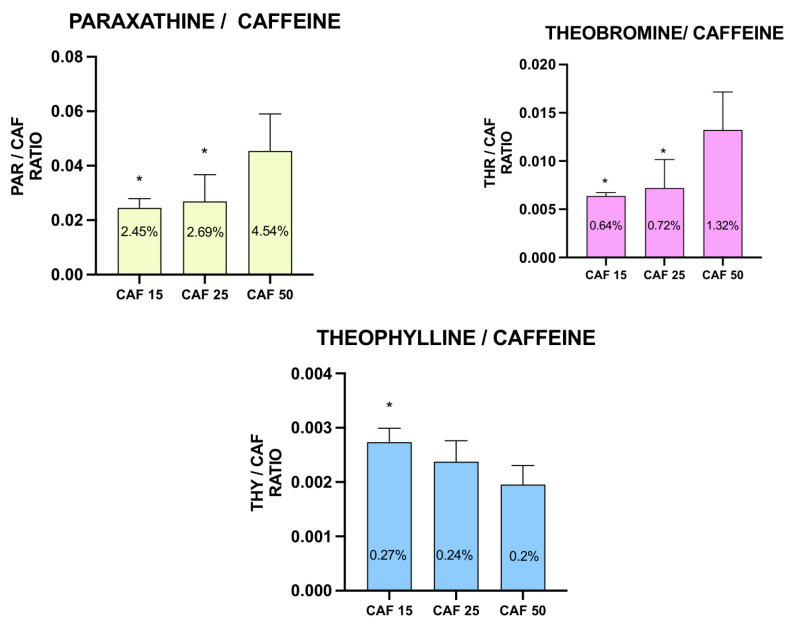
PAR/CAF, THR/CAF, and THY/CAF ratios for the larvae treated with CAF. CAF concentration applied: 15, 25, and 50 mg/L, * *p* < 0.05.

Based on these results, we assessed the ratio of CAF metabolites to CAF (Figure 3).

Similarly to PAR alone, the PAR/CAF ratio was significantly higher in the group incubated with 50 mg/L CAF compared to lower CAF doses and no difference between these lower concentrations was observed. The same relationship was observed for the THR/CAF ratio, but the ratio was lower compared to PAR. The opposite effect was observed for THY as the THY/CAF ratio decreased with higher CAF doses, and the difference was observed only between the 15 and 50 mg/L CAF groups (Figure 3). This divergent behavior indicates that THY formation may be driven by a distinct metabolic branch with limited inducibility or inhibition at higher levels of substrate.

### 2.3. CAF Metabolites Assessment

**Figure 4:**
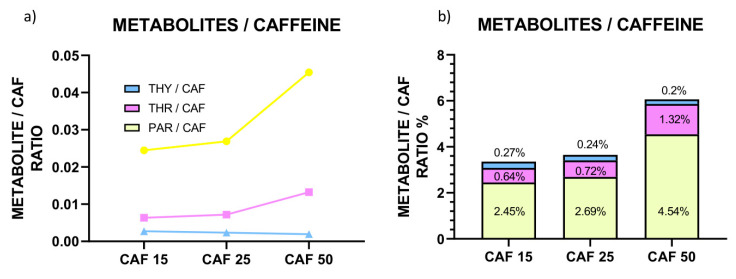
(**a**) raw values of PAR/CAF, THR/CAF, and THY/CAF ratios for the larvae treated with CAF (**b**) % of PAR/CAF, THR/CAF, and THY/CAF ratios. CAF concentration applied: 15, 25, and 50 mg/L.

Then, we summarized the data to get a full insight into the CAF metabolism (Figure 4).

As presented in Figure 4, the greater the CAF dosage, the more the drug is metabolized to PAR and THR. The most pronounced increase is observed for PAR. Inversely, the greater the CAF concentration, the less of the drug is transformed to THY (Figure 4).

**Figure 5:**
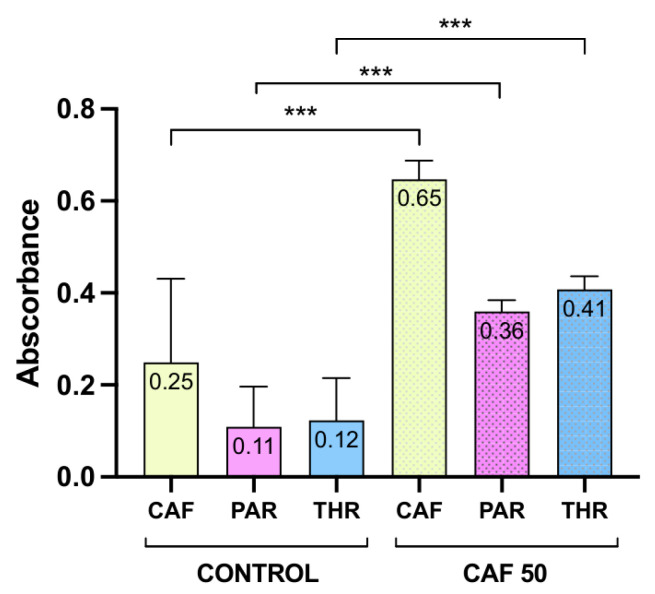
Comparison of CAF, PAR, and THR absorbance in control and CAF 50 groups; bands at 1072 and 1674 cm^−1^ were selected for CAF; 1120 and 1275 cm^−1^ for PAR and 1308 cm^−1^ for THR; *** *p* ≤ 0.0001.

Additionally, FTIR measurements were performed on metabolite standards of CAF, PAR, THR and THY. In parallel, control larvae spectra were recorded and averaged (background spectrum). Based on the evaluation of analytes and background spectrum, bands at 1072 and 1674 cm^−1^ were selected for CAF, 1120 and 1275 cm^−1^ for PAR, and 1266 and 1308 cm^−1^ for THR [16,17]. It was not possible to indicate suitable bands for THY. The mean values of absorbance for CAF, PAR, and THR (calculated as the sum of two bands) for the control and CAF 50 mg/L groups are presented in Table 2. Both CAF and its two major breakdown products were detected in the larvae’s bodies; a statistically significant difference in band intensities for CAF, PAR, and THR was observed between groups (Figure 5).

FTIR imaging was employed as a complementary approach to LC-MS/MS to provide spatial information on the distribution of CAF and its metabolites within intact zebrafish larval tissue sections. Spatial distribution data were collected as a novel approach in zebrafish studies, as presented by Kalisz et al. [18]. Notably, FTIR analysis provided spatially resolved information revealing heterogeneous patterns of CAF and its metabolites in larval tissues. This observation suggested localized metabolism or metabolite accumulation, in contrast to the low and uniform signal observed in control larvae.

**Figure 6:**
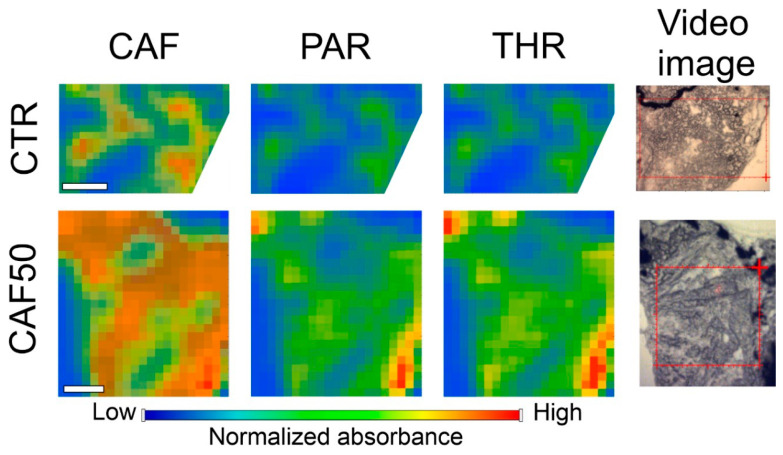
Spatial distribution of CAF and its metabolites (PAR and THR) in horizontal cross-sections of the head region mapped (red rectangles) of *Danio rerio* 5 dpf larvae. FTIR imaging in CTR and CAF50 samples, the white bar is 100 μm.

Following the spectra analysis, imaging of the head area was performed in control and CAF 50 mg/L groups, with the visualization of the abovementioned selected bands. To mitigate spectral overlap, the averaged FTIR spectrum of control larvae was used as a biological background reference and subtracted prior to map visualization, thereby removing the endogenous tissue contribution common to both groups. Presented in Figure 6, the maps show that in the control group, absorbance for all compounds is lower compared to treated larvae.

The absorbance patterns are not uniform across the treated samples, which points to localized or compartmentalized effects within the tissue (Figure 6).

## 3. Discussion

Zebrafish larvae are increasingly used in pharmacological and toxicological research due to their genetic similarity to humans and well-characterized developmental biology [19,20]. Nevertheless, the fate of CAF and its primary metabolites in this model has remained largely unexplored. Understanding CAF biotransformation in this organism is particularly relevant given that internal exposure determines the pharmacological and toxicological outcome. The present study addresses this gap by combining LC-QQQ-MS quantification with spatially resolved FTIR imaging to provide a systematic and tissue-level characterization of CAF biotransformation at the early larval stage.

Previous research indicates that the development of hepatic primordium in zebrafish starts at 28 h post fertilization (hpf), followed by hepatic outgrowth between 60 and 72 hpf. By 120 hpf, liver function, including CYP metabolism, is nearly fully established. Notably, evidence suggests that CYP metabolic functions, particularly CYP1A2, resemble those of human CYP isoforms even before complete liver development, as confirmed by both mRNA expression and metabolite analysis in larvae aged 24–120 hpf [21]. The metabolism of CAF shows considerable variability among individuals and is influenced by multiple factors, including age, gender, genetic predisposition, and environmental conditions [22]. The PAR to CAF ratio is a recognized biomarker reflecting CYP1A2 activity, with elevated ratios correlating with accelerated CAF metabolism and increased enzymatic activity of CYP1A2. Evaluating this ratio has the potential to predict individual responses to CAF and assess the propensity for adverse reactions associated with its consumption [23].

So far, only one study assessed the PAR/CAF ratio in zebrafish larvae [21]. Nawaji et al. found that only about 5% of CAF (10 mg/L) at 2 dpf and 5 dpf was transformed to PAR, suggesting that zebrafish embryos and larvae possess metabolic activity similar to that of human CYP1A2, although at a relatively low level [21]. In our study, the activity was 2.45–4.54%, depending on the dose. Our investigation revealed no statistically significant variance in the PAR/CAF ratio between larvae subjected to CAF concentrations of 15 and 25 mg/L. However, larvae treated with 50 mg/L CAF exhibited a notable elevation in the PAR/CAF ratio relative to the lower dosage cohorts. This dose-dependent increase indicates inducible or concentration-dependent activation of CYP-driven enzymatic pathways in zebrafish larvae. Nevertheless, the PAR/CAF ratio can be useful as a functional marker of metabolic activity rather than a fixed constant related to specific species.

Basnet et al. employed zebrafish embryos as a model system to investigate the overall toxicity and cardiovascular impacts of eight methylxanthines, including aminophylline, CAF, diprophylline, doxofylline, etophylline, 3-isobutyl-1-methylxanthine (IBMX), pentoxifylline, and THY [19]. They reported that CAF, IBMX, pentoxifylline, and THY induced pronounced embryotoxic and teratogenic effects. In contrast, aminophylline, doxofylline, and etophylline elicited such effects only at higher concentrations, whereas diprophylline displayed minimal (<10%) developmental toxicity. Structural abnormalities in the heart were induced by most of these drugs in 20–40% of the injected embryos at the highest dose, resulting in fatal outcomes within 120 hpf. Additionally, all drugs elicited a transient increase in heart rate at 48 hpf, which normalized by 96 hpf. This observed functional impact of methylxanthines parallels findings from studies conducted in humans and other vertebrates. Unfortunately, the referenced study did not delve into CAF metabolism; however, it did note the influence of the metabolite on the zebrafish organism [19]. In humans, CAF is reported to be transformed into PAR, THR and THY at rates of 84%, 12% and 4%, respectively [24]. In rats, CAF is demethylated to its three major metabolites in approximately equal quantities [25,26]. Our study presents that CAF in the zebrafish model is transformed into PAR, THR and THY at rates up to 4.54%, 1.32% and 0.27%, respectively. Zhao et al. conducted a study to perform a comparative transcriptome analysis of hepatocytes isolated from developing zebrafish embryos at various developmental stages (60, 72, and 96 hpf) [27]. This analysis unveiled notable alterations in gene expression profiles, elucidating pivotal biological processes and molecular pathways implicated in the proliferation and functional maturation of hepatocytes throughout liver development. These discoveries provide crucial insights into the intricate dynamics governing hepatocyte differentiation and maturation, thereby enriching our comprehension of liver development in zebrafish. In the present publication, we suggest that CAF metabolism may differ in the zebrafish model from that in the human body, which may also be important in the context of the mentioned molecular pathways. It is well known that CAF interacts with molecular cell pathways [28], which may be potentially significant for further use of the *Danio rerio* model in studies of the pathophysiology of diseases.

In this study, we also targeted CAF and metabolites using FTIR spectroscopy and spectral mapping. This technique is sensitive to changes in the secondary structure of proteins, as added chemical compounds may introduce it [29,30]. FTIR spectroscopy is a powerful tool for analyzing CAF and its metabolites. Recording their fingerprint vibrational modes enables both qualitative and semi-quantitative assessments without requiring extensive sample preparation. It provides a label-free approach in studies of molecular structures or metabolic pathways in cell biology [31]. Particularly, Ashengroph evaluated products of CAF biotransformation in an aqueous environment with *Salinvibrio costicola* [32]. Despite the fact that all metabolites were confirmed by LC-MS, only separated and purified THR was confirmed by FTIR. The important role of FTIR detection of THR and CAF was also postulated in the determination of food content, contamination [33], or crystallization [34]. The analysis of PAR and THY was less reported, possibly due to problems with low concentration and partially overlapping spectral features in metabolism studies. THY is also studied with FTIR as an active pharmaceutical ingredient [35]. Spectral overlap in the fingerprint region represents a general analytical challenge in biological FTIR imaging; proteins (Amide III, 1230–1300 cm^−1^), nucleic acids (νas/νs PO_2_^−^, 1080–1240 cm^−1^), carbohydrates (C–O–C stretching, 1030–1080 cm^−1^), and lipids (C–O stretching, 1050–1100 cm^−1^) all absorb in the same fingerprint region used for methylxanthine detection, which likely contributes to the limited reporting of PAR and THY in tissue-imaging studies. While FTIR has been successfully used in pharmaceutical and microbial systems, its application in higher model organisms, such as zebrafish larvae, remains underexplored. Spatially resolved data coupling zebrafish larvae and FTIR imaging is a novel approach, as only one report on the distribution of amides and lipids was reported in the available literature [18]. Our study addresses this gap by providing information on localized CAF biotransformation with visualization in larvae. This approach complements traditional methods such as liquid chromatography–tandem mass spectrometry (LC-QQQ-MS) and offers insights into molecular changes in models. Future studies could focus on improving the sensitivity for detecting low-abundance metabolites and the application of spatially resolved FTIR imaging in model organisms that could further validate its utility in metabolic studies, as a part of the “spectralomic” approach [31].

Comprehending CAF metabolism and the involvement of cytochrome P-450 is crucial for anticipating individual reactions to CAF and identifying variables that may impact CAF metabolism. This knowledge can inform strategies for the prudent and efficient utilization of CAF-containing products, particularly in individuals concurrently taking medications that interact with CAF. Furthermore, research indicates that a higher CAF metabolite ratio may be correlated with a reduced risk of specific health ailments, such as Parkinson’s disease, whereas a lower ratio may be associated with heightened susceptibility to cardiovascular complications.

The present study focused on the three primary CAF metabolites known from mammalian systems (PAR, THR, and THY). Due to the targeted nature of the LC-MS/MS method applied, the analysis was limited to predefined compounds. However, the formation of additional minor metabolites in zebrafish larvae cannot be excluded and would require future studies for comprehensive characterization.

## 4. Materials and Methods

### 4.1. Animals

Wild-type zebrafish (*Danio rerio*) of the AB strain (Experimental Medicine Centre, Medical University of Lublin, Poland) were housed under controlled conditions at temperatures between 26 and 28.5 °C. Water pH was maintained in the range of 6.9 to 7.5, conductivity values were kept between 550 and 700 µS/cm, and a 14/10 h light-to-dark photoperiod was applied. Following fertilization, embryos were kept in E3 embryo medium and incubated under standard light/dark conditions using an IN 110 incubator (Memmert GmbH, Buechenbach, Germany). Larvae at 4 days post-fertilization (dpf) were selected for use in all experimental procedures. Upon completion of the experiments, larvae were euthanized by immersion in ice-cold water at a ratio of 5 parts ice to 1 part water (0 to 4 °C) for 20 min. Subsequently, specimens were frozen at −22 °C to ensure cessation of brain activity. All procedures were carried out in full compliance with the National Institute of Health Guidelines for the Care and Use of Laboratory Animals. The European Community Council Directive 2010/63/EU of 22 September 2010 was also followed. As the study involved larvae at a developmental stage not exceeding 5 dpf, approval from the Local Ethical Commission was not required.

### 4.2. Chemicals

CAF, PAR, THR, and THY were obtained from Sigma-Aldrich (Saint Louis, MO, USA) at pharmaceutical secondary standards with a purity > 99% (LC-MS grade). Stock solutions were prepared by dissolving each compound in deionized water. The solutions were subsequently diluted in E3 embryo medium to reach the desired final concentrations. The E3 medium was composed of 17.4 μM NaCl, 0.21 μM KCl, 0.12 μM MgSO_4_ and 0.18 μM Ca(NO_3_)_2_, with pH adjusted to a range of 7.1 to 7.3.

### 4.3. Treatments for the Assessment of CAF and Its Metabolites Concentrations

A total of five larvae per well were placed in a 48-well plate containing 500 μL of either E3 embryo medium or CAF at concentrations of 15, 25, and 50 mg/L. Preincubation was carried out at 28 °C for 18 h. The applied CAF concentrations and exposure duration were established based on the available literature and previous research conducted in a home laboratory using zebrafish larvae as a model organism [36,37,38,39].

### 4.4. Sample Preparation

Larvae were rinsed three times with E3 embryo medium and subsequently transferred to tubes at a density of 20 larvae per sample with 5 samples per experimental group. Tissue homogenization was performed by sonication (4 cycles of 5 s each) in 150 μL of 100 mM NH_4_HCO_3_. The homogenate was then kept on ice for 15 min and centrifuged at 15,000 *g* for 15 min at 4 °C. A volume of 100 μL of the resulting supernatant was combined with a methanol and ethanol solution (1:1) to achieve a final ratio of 1:3. The mixture was vortexed for 30 s and incubated at 20 °C for 15 min. A subsequent centrifugation step was performed at 15,000 *g* for 10 min at 4 °C. Finally, 250 μL of the obtained supernatant was transferred to a chromatography vial.

### 4.5. LC-QQQ-MS Analysis

Chromatographic separation was carried out with the Agilent 1290 Infinity II LC system coupled to the Agilent 6470 Triple Quad tandem mass spectrometer equipped with an electrospray ionization source, Jet Stream Technology (Agilent Technologies, Santa Clara, CA, USA). The chromatographic separations were conducted using an RRHD Zorbax Eclipse Plus C18 column (Agilent Technologies; 2.1 × 100 mm; 1.8 μm) at a flow rate of 0.3 mL/min. The mobile phases were 0.1% formic acid in water (A) and 0.1% formic acid in methanol (B). The run time was 7 min, with the gradient program 0–4 min, 13–18% B; 4.01–6 min, 60–75% B; 6.01–7 min, 95% B and final column conditioning of 3 min. The injection volume was set to 2 μL, and the column temperature was held at 40 °C. The mass spectrometer was operated in positive electrospray ionization mode (ESI+) with the following settings: ion source gas, N_2_; ion source gas temperature, 300 °C; ion source gas flow rate, 12 L/min; nebulizer pressure, 40 psi; sheath gas temperature, 350 °C; sheath gas flow rate, 12 L/min; and capillary voltage, 4000 V. The needle was washed after each injection with a mixture of methanol and water (80:20, *v*/*v*). Full acquisition details and optimized MS parameters are provided in Table 1.

Data acquisition was carried out using Agilent Mass Hunter Data Acquisition software (version B.09.00). Subsequent data processing was performed with Agilent Mass Hunter Qualitative Analysis software (version B.10.02) (Agilent Technologies, Santa Clara, CA, USA). The amount of CAF and its three primary metabolites, PAR, THR, and THY, in control larvae was determined from the corresponding matrix-matched calibration curve. The linear ranges of calibration curves were estimated using the coefficient of determination (R^2^ ≥ 0.99). LOD and LOQ values were derived from signal-to-noise ratios of 3:1 and 10:1, respectively. Calculations were based on the most intense MRM transition recorded at the lowest concentration level of the calibration sample. Concentration results were reported as mean values across five injections per group.

### 4.6. Fourier Transform Infrared Spectroscopy

Fourier transform infrared spectroscopy (FTIR) measurements were carried out in the reflection mode using an FTIR spectrometer (Nicolet 8700, Thermo, Waltham, MA, USA) attached to an infrared microscope (Nicolet Continuum, Thermo, Waltham, MA, USA) with an MCT-A detector. The microscope was equipped with a ×15 IR objective. Spectra within the 4000–650 cm^−1^ spectral range were measured with 120 scans at 8 cm^−1^ spectral resolution using Happ–Genzel apodization and two levels of zero filling. Area maps were acquired in automated point-mapping mode with a stage step size of 20 × 20 µm and an aperture of 25 µm, yielding a spatial resolution of 20 µm per pixel. Background spectra were collected from a region free of tissue using 120 scans. Frozen larvae from control and CAF 50 mg/L were subjected to horizontal sectioning with Leica 19650UV (Leica Microsystems, Wutzlar, Germany) to 10 μm slices embedded in gelatin from porcine skin (Sigma-Aldrich, St. Louis, MO, USA) and placed on reflective, aluminum-coated slides. The assessed physical characteristics of the samples (size and thickness) were standardized for the investigations. Initially, the FTIR spectra for CAF, PAR, THR, and THY standards (Sigma Aldrich) were recorded concurrently with 100 spectra randomly collected from control and CAF 50 mg/L larvae whole bodies. Finally, in imaging mode, the head area measurements were carried out step-by-step, with a 20 µm step in the x/y plane.

FTIR spectral data were analyzed using the OMNIC 8 software (8.2.0.387 Thermo Fisher Scientific, Waltham, MA, USA) and Orange (ver. 3.36.2, University of Ljubljana, Ljubljana, Slovenia) software. The data were preprocessed with algorithmic baseline correction with a rubber band algorithm, Gaussian smoothing (SD: 5) and vector normalization. The absorbance intensity was used for statistical evaluation of larvae spectral data.

### 4.7. Statistical Analysis

All statistical analyses were carried out in GraphPad Prism 8 (GraphPad Software, San Diego, CA, USA). Depending on the experimental design, one-way or two-way ANOVA was applied. One-way ANOVA was followed by Tukey’s post hoc test. A *p*-value < 0.05 was considered statistically significant. Results are expressed as mean ± standard deviation (SD). Larvae were randomly assigned to experimental groups prior to the start of the experiment.

## 5. Limitations

This study provides novel insights into CAF biotransformation in zebrafish larvae; however, several limitations should be acknowledged. First, the applied LC-MS/MS approach was based on targeted analysis, focusing on CAF and its three primary metabolites (PAR, THR and THY) known from mammalian systems. Consequently, the presence of additional minor or alternative metabolites cannot be excluded and would require untargeted metabolomics approaches for comprehensive characterization. FTIR imaging was carefully performed using two bands per analyte and the mean control spectrum as background to reduce the risk of false attribution; however, complete spectral separation was not achieved. It was not possible to identify analytically suitable bands for THY, which was therefore excluded from FTIR mapping. Collectively, FTIR-derived band intensities should be interpreted as semiquantitative indicators of spatial distribution, complementary to the LC-MS/MS quantification data. The study was conducted at a single developmental stage (4–5 dpf), which represents a period of ongoing physiological maturation. Therefore, the observed metabolic activity may not fully reflect the metabolic capacity of later developmental stages or adult organisms.

## 6. Conclusions

We conducted a comprehensive investigation into CAF metabolism in zebrafish larvae, focusing on its three primary metabolites: PAR, THY, and THR. To characterize this biotransformation quantitatively, a validated LC-QQQ-MS method was developed and applied for the simultaneous determination of CAF and its primary metabolites, complemented by FTIR imaging for spatially resolved metabolite mapping in larval tissues. The quantitative method achieved LOD values of 5.358, 0.430, 1.118, and 0.389 nmol/L and LOQ values of 17.859, 1.434, 3.725, and 1.298 nmol/L for CAF, PAR, THR, and THY, respectively, with precision (RSD) not exceeding 9.01%. Zebrafish larvae exposed to CAF at 15, 25, and 50 mg/L revealed dose-dependent biotransformation: CAF was converted to PAR at up to 4.54%, to THR at up to 1.32%, and to THY at up to 0.27% of the administered dose. Measured internal CAF concentrations reached 504.7, 692.2, and 1444.7 nmol/L, while PAR, THR, and THY peaked at 65.3, 19.0, and 2.8 nmol/L, respectively, at the highest exposure level. Complementary FTIR imaging confirmed the presence of CAF, PAR, and THR in larval tissues and revealed their heterogeneous spatial distribution, with statistically significant differences in absorbance between control and CAF 50 mg/L groups. To our knowledge, this study is the first to assess CAF metabolites in zebrafish larvae, offering a novel analytical approach applicable to various zebrafish-related research endeavors. Our findings underscore the presence of CYP450-mediated demethylation activity in zebrafish larvae before full liver development, highlighting the significance of early-stage zebrafish studies in pharmacological research. Further investigations involving older larvae and adult fish are warranted to elucidate the complete spectrum of CAF metabolism in zebrafish. The integration of quantitative LC-MS/MS methods with spatially resolved FTIR imaging described in this study contributes to the development of the zebrafish model by extending its application towards more mechanistically informed approaches for potential xenobiotic investigation of metabolism and tissue-specific biochemical responses.

## Figures and Tables

**Table 1:** Multiple reaction monitoring parameters for caffeine and its primary metabolites, obtained using LC-QQQ-MS in positive electrospray ionization mode.

Analyte	Formula	RT (min)	RT Window (min)	Precursor Ion *m*/*z*	Product Ion *m*/*z*	Frag. (V)	CE (V)	LOD (nmol/L)	LOQ (nmol/L)
Caffeine	C_8_H_10_N_4_O_2_	5.29	0.5	195.1	110	136	25	5.358	17.859
					138	136	21		
					83	136	29		
					69.1	136	33		
					56.1	136	37		
Paraxanthine	C_7_H_8_N_4_O_2_	3.33	0.5	181.1	124	118	22	0.430	1.434
					96	118	26		
					69.1	118	38		
					67	118	42		
					55.1	118	34		
Theobromine	C_7_H_8_N_4_O_2_	2.28	0.5	181.1	138	121	18	1.118	3.725
					110.1	121	22		
					83.1	121	30		
					67.1	121	38		
Theophylline	C_7_H_8_N_4_O_2_	3.68	0.5	181.1	124	103	22	0.389	1.298
					94	103	26		
					69.1	103	34		

LOD—limit of detection; LOQ—limit of quantification; RT—retention time; Frag—fragmentor; CE—collision energy.

**Table 2:** Mean absorbance intensity of peaks assigned to CAF, PAR, and THR, in 5 dpf zebrafish larvae ± SD, *** *p* ≤ 0.0001.

Treatment	CAF	PAR	THR
CTR	0.25 ± 0.18 ***	0.11 ± 0.09 ***	0.12 ± 0.09 ***
CAF 50	0.65 ± 0.40 ***	0.36 ± 0.25 ***	0.41 ± 0.29 ***

## Data Availability

The raw data supporting the conclusions of this article will be made available by the authors, without undue reservation.

## Abbreviations

The following abbreviations are used in this manuscript:

ANOVA	Analysis of variance
ARRIVE	Animal Research: Reporting of In Vivo Experiments
ATR-FTIR	Attenuated Total Reflectance Fourier-Transform Infrared Spectroscopy
CAF	Caffeine
CE	Collision energy
CTR	Control group
CYP	Cytochrome P450
CYP1A2	Cytochrome P450 1A2
dMRM	Dynamic Multiple Reaction Monitoring
dpf	Days post-fertilization
E3	Embryo medium (E3 medium)
ESI+	Electrospray ionization, positive mode
FTIR	Fourier-transform infrared spectroscopy
hpf	Hours post-fertilization
LC	Liquid chromatography
LC-MS	Liquid chromatography–mass spectrometry
LC-MS/MS	Liquid chromatography–tandem mass spectrometry
LC-QQQ-MS	Liquid chromatography–triple quadrupole mass spectrometry
LOD	Limit of detection
LOQ	Limit of quantification
MCT-A	Mercury Cadmium Telluride Detector (Type A)
MRM	Multiple reaction monitoring
PAR	Paraxanthine
RSD	Relative standard deviation
RT	Retention time
SD	Standard deviation
THR	Theobromine
THY	Theophylline
UV	Ultraviolet
